# The Etiology of Science Performance: Decreasing Heritability and Increasing Importance of the Shared Environment From 9 to 12 Years of Age

**DOI:** 10.1111/j.1467-8624.2009.01289.x

**Published:** 2009-05

**Authors:** Claire M A Haworth, Philip S Dale, Robert Plomin

**Affiliations:** Institute of Psychiatry, King’s College London; University of New Mexico; Institute of Psychiatry, King’s College London

## Abstract

During childhood and adolescence, increases in heritability and decreases in shared environmental influences have typically been found for cognitive abilities. A sample of more than 2,500 pairs of twins from the Twins Early Development Study was used to investigate whether a similar pattern would be found for science performance from 9 to 12 years. Science performance was based on teacher-assessed U.K. National Curriculum standards. Science at 9 years showed high heritability (64%) and modest shared environmental (16%) estimates. In contrast to the expected developmental pattern, heritability was significantly lower at 12 years (47%) and shared environmental influences were significantly higher (32%). Understanding what these increasingly important shared environmental influences are could lead to interventions that encourage engagement in science throughout the lifespan.

The development of scientific knowledge and reasoning is an important goal in a society that places great importance on the study and application of science and technology (HM Treasury/DTI/DfES, 2005). Although science is not one of the core educational domains—reading, writing, and arithmetic—it has increasingly become a focus for education. For example, in the United Kingdom, science became a compulsory subject in elementary teaching in 1989 with the introduction of the National Curriculum (NC). In addition, public engagement and understanding of science throughout the lifespan is becoming more important in our increasingly technological world (British Association for the Advancement of Science, 2005; Osborne & Dillon, 2008). An understanding of the genetic and environmental origins of science performance throughout the school years has implications for educational policy and educational interventions (Grigorenko, 2007; Haworth, Meaburn, Harlaar, & Plomin, 2007; Plomin, Kovas, & Haworth, 2007). Behavioral genetic research into academic abilities has focused on reading and more recently mathematics (e.g., Harlaar, Hayiou-Thomas, & Plomin, 2005; Kovas, Petrill, & Plomin, 2007). Far less attention has been given to science, and in particular to the developmental etiology of science performance.

## Previous Twin Studies of Science

Genetic research on science performance has used the twin method that compares the resemblance of identical and fraternal twin pairs. The first twin study of science performance was conducted in the present sample when the twins were 9 years old. Genetic influences accounted for more than 60% of the variance, and shared environmental influences accounted for 14% of the variance (Haworth, Dale, & Plomin, 2008). At 9 years there was no evidence for sex differences in means or in genetic and environmental influences on the variance. Based on this study, the etiology of science performance at 9 years is highly comparable with the etiology of mathematics and English performance at this age, which both show high heritability and modest influence of the shared environment (e.g., Kovas, Haworth, Dale, & Plomin, 2007). For science-related abilities, the only other genetic study is a twin study of bright high school students that reported heritability of 40% and shared environment of 30% for a measure of critical reading of scientific material (Loehlin & Nichols, 1976).

## Developmental Trends in the Etiology of Cognitive and Academic Abilities

Developmental analyses of twin study data, both cross-sectional and longitudinal, are available for general cognitive ability (g), reading, and math. The present study is the first to consider the developmental etiology of science performance. Genetic research on g in particular has yielded two developmental trends. First, heritability increases linearly from about 20% in infancy to about 40% in middle childhood, and to about 50% in adolescence and young adulthood, and even higher in middle age (Boomsma, 1993; McGue, Bouchard, Iacono, & Lykken, 1993; Plomin, 1986). The cause of this developmental increase in heritability is not known but one possibility is that as children increasingly make their own way in the world they move from experiencing environments largely created by other people to actively creating correlations between their genetic propensities and their experiences (Plomin & DeFries, 1985; Scarr, 1968). Second, shared environmental influence decreases sharply from about 30% in childhood to near 0% in adolescence, perhaps as adolescents increasingly live their lives outside their family. (Note that because there is a third category of influence—nonshared environment—these results concerning heritability and shared environment are empirically independent findings.) To the extent that academic achievement reflects g, similar developmental trends would be expected for academic performance.

Although there are few developmental studies of the etiology of academic abilities, and their measures and samples differ considerably, the results suggest a trend in the same direction. Outside of Twins Early Development Study (TEDS), the only study of academic performance in the early school years yielded estimates of moderate heritability and moderate shared environment (about 40%) for overall academic performance (Thompson, Detterman, & Plomin, 1991). In early adolescence there is an increase in heritability and a decrease in shared environmental influences for academic performance: Two studies yielded average estimates of about 55% heritability and 30% shared environment (Bartels, Rietveld, van Baal, & Boomsma, 2002; Husén, 1959). Similar results were found in studies of late adolescence: Two studies yielded average estimates of about 50% heritability and 20% shared environment (Loehlin & Nichols, 1976; Wainwright, Wright, Geffen, Luciano, & Martin, 2005; Wainwright, Wright, Luciano, Gelfen, & Martin, 2005).

Among specific academic abilities, reading has attracted the most attention. In the Colorado Adoption Project, word recognition was examined longitudinally in a sample of adoptive and nonadoptive sibling pairs (Wadsworth, Corley, Hewitt, & DeFries, 2001; Wadsworth, Corley, Plomin, Hewitt, & DeFries, 2006). Heritability increased from 7 to 16 years (44% and 57%) and genes were largely responsible for the substantial stability from age to age. Shared environmental influences remained consistently low (7% and 7%) and nonshared environmental influences decreased from 7 to 16 years (49% and 36%). These rare data for adoptive and nonadoptive siblings are especially important because, unlike twin analyses, adoptive sibling correlations provide a direct test of the shared environmental influence. Another longitudinal twin study of early reading from kindergarten to first grade (Byrne et al., 2005; Byrne et al., 2007) also found increases in heritability and decreases in shared environmental influences during early development. Results for reading from the TEDS follow a similar pattern; the heritability of reading ability in TEDS remains high from 7 (65%) to 10 (67%) years (Harlaar, Dale, & Plomin, 2007). We have also investigated the developmental etiology of mathematics performance (Haworth, Kovas, Petrill, & Plomin, 2007; Kovas, Petrill, et al., 2007): From 7 to 9 years, heritability for mathematics performance increased (62% and 71%), and genetic influences explained 81% of the cross-age continuity (Haworth, Kovas, et al., 2007). At 7 and 9 years, shared environmental influences on mathematics performance were nonsignificant (Haworth, Kovas, et al., 2007; Oliver et al., 2004).

Thus, results from longitudinal studies to date indicate that heritability for academic traits such as reading and mathematics remains high throughout development, with some indication that like g, heritability estimates increase during development. However, in most of these cases any changes across time are not statistically significant.

## The Present Study

We aimed to investigate the developmental etiology of school science performance from 9 to 12 years. We have previously shown that science performance is highly heritable at 9 years, and that shared environmental influences make only a modest contribution (Haworth et al., 2008). Based on previous studies of other academic abilities, we predicted that the heritability of science performance would remain high at 12 years, perhaps even increasing, and that the shared environment would make only a modest contribution. In addition, we predicted that genetic influences would explain most of the continuity in performance across time, consistent with previous longitudinal twin studies of reading and mathematics performance.

## Method

### Sample

The sampling frame for the present study was the TEDS, a study of twins born in England and Wales in 1994, 1995, and 1996 (Oliver & Plomin, 2007; Trouton, Spinath, & Plomin, 2002). The TEDS sample has been shown to be reasonably representative of the general population in terms of parental education, ethnicity, and employment status (Kovas, Petrill, et al., 2007). For example, in the U.K. census data (2001), 92% of the population was White compared with 92% of the TEDS sample. Zygosity was assessed through a parent questionnaire of physical similarity, that has been shown to be over 95% accurate when compared with DNA testing (Price et al., 2000). For cases where zygosity was unclear from this questionnaire, DNA testing was conducted.

Twins born in 1994 and 1995 were invited to participate in the study at age 9. At age 12, twins born in 1994, 1995, and 1996 were invited to participate. As described below, science performance was assessed by teacher ratings based on the U.K. NC criteria. Of the teacher rating forms sent at 9 years, 5,836 individual forms were returned complete (76% of those contacted). At age 12, 9,905 individual forms were returned complete (78%). Not all teachers were able to provide NC levels, and for some twin pairs we only received a completed form for one member of the pair. In Tables 1 and 2 we present the sample sizes for the science measures at each age.

**Table 1:** Means (*Standard Deviations*) by Zygosity and Sex and ANOVA Results Showing Significance and Effect Size by Zygosity and Sex

		Zygosity		Sex		ANOVA		
Measure	All	MZ	DZ	Female	Male	Zygosity	Sex	Zygosity × Sex
9 year								
Scientific enquiry	2.96 (.63), *n* = 2,729	2.92 (.63), *n* = 976	2.98 (.62), *n* = 1,753	2.93 (.60), *n* = 1,440	2.98 (.65), *n* = 1,289	*p* = .015, *η*^2^ = 0.002	*p* = .182, *η*^2^ = 0.001	*p* = .166, *η*^2^ = 0.001
Life processes	3.01 (.59), *n* = 2,713	2.99 (.59), *n* = 971	3.03 (.59), *n* = 1,742	3.00 (.56), *n* = 1,433	3.02 (.62), *n* = 1,280	*p* = .121, *η*^2^ = 0.001	*p* = .615, *η*^2^ < 0.001	*p* = .400, *η*^2^ < 0.001
Physical processes	2.98 (.60), *n* = 2,693	2.96 (.60), *n* = 961	2.99 (.60), *n* = 1,732	2.96 (.58), *n* = 1,422	3.00 (.63), *n* = 1,271	*p* = .199, *η*^2^ = 0.001	*p* = .199, *η*^2^ = 0.001	*p* = .310, *η*^2^ < 0.001
12 year								
Scientific enquiry	4.39 (.98), *n* = 3,801	4.35 (.96), *n* = 1,376	4.41 (1.00), *n* = 2,425	4.36 (.96), *n* = 2,009	4.42 (1.01), *n* = 1,792	*p* = .041, *η*^2^ = 0.001	*p* = .190, *η*^2^ < 0.001	*p* = .258, *η*^2^ < 0.001
Life processes	4.50 (.96), *n* = 3,762	4.46 (.94), *n* = 1,360	4.52 (.97), *n* = 2,402	4.49 (.93), *n* = 1,988	4.51 (.99), *n* = 1,774	*p* = .052, *η*^2^ = 0.001	*p* = .741, *η*^2^ < 0.001	*p* = .532, *η*^2^ < 0.001
Physical processes	4.46 (.96), *n* = 3,749	4.41 (.94), *n* = 1,360	4.48 (.98), *n* = 2,389	4.44 (.94), *n* = 1,983	4.48 (.99), *n* = 1,766	*p* = .040, *η*^2^ = 0.001	*p* = .365, *η*^2^ < 0.001	*p* = .473, *η*^2^ < 0.001
Materials and their properties	4.47 (.96), *n* = 3,761	4.42 (.95), *n* = 1,363	4.50 (.96), *n* = 2,398	4.45 (.94), *n* = 1,993	4.49 (.98), *n* = 1,768	*p* = .017, *η*^2^ = 0.002	*p* = .440, *η*^2^ < 0.001	*p* = .232, *η*^2^ < 0.001

*Note.* MZ = monozygotic; DZ = dizygotic; *η*^2^ = effect size; ANOVA = analysis of variance. ANOVA and means for one randomly selected member of each twin pair (*n* indicates number of randomly selected individuals).

For the purposes of the current study, we excluded 697 individuals from the analysis because at least one member of the twin pair had a specific medical syndrome or was an extreme outlier for perinatal problems such as extreme low birth weight. At age 9, the mean age of the twins participating in the study was 9.04 (*SD* = .29) and at age 12 the mean age of the twins taking part in the study was 11.54 (*SD* = .66).

### Measures

As for all children in the United Kingdom, the twins’ academic performance was assessed throughout the school year by their teachers using the assessment materials of the NC for England and Wales, the core academic curriculum developed by the Qualifications and Curriculum Authority (QCA). Teachers were contacted when the children were in the second half of their school year so that the teachers would be familiar with the children’s performance during the school year. Teachers were sent a cover letter with the background and aims of TEDS, as well as explaining that we had obtained consent from the twins’ parents to ask teachers for information about the child’s performance at school.

For the study at age 9, the NC Teacher Assessments at Key Stage 2 were used, that are familiar to teachers and were designed for children in their 3rd year of primary school at age 8 through their 6th year of primary school at age 11. Teachers assessed three broad areas of science performance: scientific enquiry, life processes and living things, and physical processes. For the study at age 12, the NC Teacher Assessments at Key Stage 3 were used, which were designed for children aged 11–14 years old. When the twins were 12 years old, teachers assessed four broad areas of science ability: scientific enquiry, life processes and living things, physical processes, and materials and their properties. Teachers rate performance from Levels 1 to 8. By the end of Key Stage 2 (age 11), children are expected to reach Level 4, and by the end of Key Stage 3 at age 14, children are expected to reach Levels 5 or 6.

For scientific enquiry, example criteria for Level 3 (which was the mean level of performance of TEDS twins at age 9) are: (a) Pupils respond to suggestions and put forward their own ideas about how to find the answer to a question. (b) Pupils recognize why it is important to collect data to answer questions. (c) They make relevant observations and measure quantities such as length or mass using a range of simple equipment. At 12 years the TEDS twins were performing between Levels 4 and 5 on average. For Level 4, criteria for scientific enquiry include: (a) Pupils recognize that scientific ideas are based on evidence. (b) In their own investigative work, they decide on an appropriate approach (for example, using a fair test) to answer a question. (c) They begin to plot points to form simple graphs, and use these graphs to point out and interpret patterns in their data. For Level 5, criteria for scientific enquiry include: (a) Where appropriate, pupils make predictions based on their scientific knowledge and understanding. (b) They begin to repeat observations and measurements and to offer simple explanations for any differences they encounter. (c) They draw conclusions that are consistent with the evidence and begin to relate these to scientific knowledge and understanding. (d) They make practical suggestions about how their working methods could be improved. Additional details about these measures have been published previously (Haworth et al., 2008; Kovas, Petrill, et al., 2007) and further information about the other domains of science in the NC as well as examples of work at different levels are available at http://curriculum.qca.org.uk.

These judgments were not made specifically for the present study but rather form the continuing assessment of each child that ultimately leads to the final NC Teacher Assessment score submitted to the QCA at the end of the key stage that summarized the child’s academic achievement during that period. Reminders of the NC criteria used to select the appropriate attainment level were provided as part of the questionnaire. The science scales were highly correlated, with an average intercorrelation of .85 at 9 years, and .93 at 12 years. A factor analysis indicated that the first principal component accounted for 90% of the variance at 9 years and 95% of the variance at 12 years.

### Data Preparation and Phenotypic Analyses

Analysis of variance (ANOVA) was used to analyze sex differences in means and variances of the raw science scores. Most of the analyses focus on the etiological analysis of individual differences using the classical twin method. For these etiological analyses, all measures were standardized to 0 *M* and 1 *SD* on the basis of the entire sample of twins (with children with major perinatal and medical problems excluded as described earlier). Because twins were perfectly correlated for age and same-sex twins are correlated perfectly for sex, variation associated with age or sex would contribute to the correlation between twins. That is, data uncorrected for age and sex would inflate twin correlations. For this reason, and as is standard in twin analysis, all measures were corrected for age and sex effects using a regression procedure (McGue & Bouchard, 1984). The proportion of the variance for a particular trait that is attributable to additive genetic influences and shared and nonshared environmental influences can be estimated from twin analyses. The sample included both male and female same-sex pairs as well as opposite-sex fraternal twin pairs. At 9 years, we found no significant sex differences in genetic or environmental etiology (Haworth et al., 2008). Similar results were found at 12 years (details available from first author), and therefore we report results here from analyses with the sexes combined, including opposite-sex as well as same-sex twins.

### The Twin Method

The twin method uses MZ (monozygotic, identical) and DZ (dizygotic, fraternal) twin intraclass correlations to dissect phenotypic variance into genetic and environmental sources (Plomin, DeFries, McClearn, & McGuffin, 2008). MZ twins are 100% genetically similar, whereas DZ twins are on average only 50% similar for segregating genes. Environmental variance can be dissected into shared environmental effects (i.e., environmental effects that make members of the same family more similar) and nonshared environmental effects (i.e., environmental effects that do not make members of the same family similar). These genetic and environmental effects, are commonly known as A, C, and E. The A component is the additive genetic effect size, also known as narrow heritability. Heritability can be estimated by doubling the difference between MZ and DZ twin correlations. For example, if MZ and DZ twin correlations are .80 and .50, respectively, heritability (A) is estimated as 60%. Shared environment (C, for effects in common to family members) refers to variance that makes MZ and DZ twins similar beyond twin similarity explained by additive genetic effects. C can be estimated by subtracting the estimate of heritability from the MZ correlation. In the earlier example, the C component would be estimated as 20%. In addition, nonshared environmental influences (E) can be estimated from the total variance not shared by MZ twins; nonshared environmental influences are the only influences deemed to make MZ twins different. Therefore, in the earlier example, the E component would be estimated as 20%, that is 100%–80%. The total variance explained cannot exceed 100% (Plomin et al., 2008). E also includes measurement error.

### Analyses

Twin intraclass correlations were calculated that index the proportion of total variance due to between-pair variance (Shrout & Fleiss, 1979). Rough estimates of genetic and environmental influences were calculated from these twin correlations. A more elegant way of estimating the ACE parameters is maximum-likelihood model-fitting analysis (Plomin et al., 2008) that provides estimates of genetic and environmental effect sizes that made assumptions explicit, tests the fit of the entire model to the data, tests the relative fit of alternative models, and provides confidence intervals for the parameter estimates. Discussion of the use of maximum-likelihood model-fitting analyses can be found elsewhere (Neale, Boker, Xie, & Maes, 2006; Neale & Maes, 2001; Plomin et al., 2008; Rijsdijk & Sham, 2002). Mx software for structural equation modeling was used to perform standard model-fitting analyses (Neale et al., 2006).

For the model-fitting analyses, we used a bivariate common pathway model (Neale & Maes, 2001). This model includes a phenotypic factor analysis of the different science measures at each age, which is the basis for creating a latent science variable. The variance of these latent variables (one at 9 years and one at 12 years) is then decomposed into genetic and environmental influences. By decomposing the covariance between the latent science variables at 9 and 12 years in a similar fashion, it is possible to estimate the genetic and environmental correlations between 9 and 12 years, that is, the extent to which genes that influence science at 9 years also influenced science at 12 years, and similarly for environmental influences. In addition, specific genetic and environmental factors are calculated for the remaining variance for each of the science measures (i.e., the variance not included in the latent variable). To assess changes in parameter estimates from 9 to 12 years we also ran a univariate common pathway model for age 9 and then for age 12, and then compared the fit when we equated the ACE parameters across the models.

## Results

The means and standard deviations for all the measures at 9 and 12 years are presented in Table 1. The results of a 2 × 2 (Sex × Zygosity) ANOVA, also shown in Table 1, indicate no significant effects of sex on the science measures at 9 and 12 years. Although there were significant main effects of zygosity, these significant effects were very small, generally accounting for less than 1% of the variance. There were no significant interactions between sex and zygosity. The mean NC levels at ages 9 and 12 were consistent with the expected level of achievement for these age groups.

### Intraclass Twin Correlations

The twin intraclass correlations for all the measures are shown in Table 2, presented for MZ twins and DZ twins. In every case, MZ correlations exceeded those of the DZ twins, suggesting genetic influence. For the entire sample, doubling the difference between the MZ and the DZ correlations to estimate heritability indicated that at 9 years genetics substantially influenced science performance (average = .61). Estimates of the shared environment—subtracting the above estimates of heritability from the MZ twin correlation—were moderate at 9 years (average = .13). At 12 years heritability was lower (average = .37) and shared environmental influences were higher (average = .36). The heritability (A), shared environment (C), and nonshared environment (E) estimates that were calculated from the intraclass correlations are included in Table 2.

**Table 2:** Twin Intraclass Correlations and ACE Estimates Derived From the Twin Correlations

Measure	MZ	DZ	A	C	E
9 year					
Scientific enquiry	0.73 (*n* = 910)	0.45 (*n* = 1,626)	.56	.17	.27
Life processes	0.75 (*n* = 901)	0.43 (*n* = 1,609)	.64	.11	.25
Physical processes	0.74 (*n* = 893)	0.43 (*n* = 1,596)	.62	.12	.26
12 year					
Scientific enquiry	0.71 (*n* = 1,175)	0.55 (*n* = 1,980)	.32	.39	.29
Life processes	0.73 (*n* = 1,152)	0.53 (*n* = 1,955)	.40	.33	.27
Physical processes	0.73 (*n* = 1,148)	0.55 (*n* = 1,936)	.36	.37	.27
Materials and their properties	0.73 (*n* = 1,158)	0.53 (*n* = 1,958)	.40	.33	.27

*Note.* MZ = monozygotic; DZ = dizygotic; A = additive genetic; C = shared environment; E = nonshared environment. All significant at .01 alpha level. *n* indicates number of complete twin pairs.

### Model-Fitting Analyses

**Figure 1:**
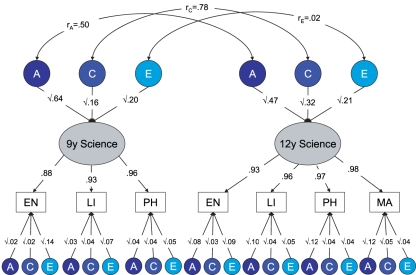
Common pathway model for science performance from 9 to 12 years. *Note*. EN= scientific enquiry; LI = life processes; PH = physical processes; MA = materials and their properties; A = additive genetic; C = shared environment; E = nonshared environment; *r*_A_ = genetic correlation; *r*_C_ = shared environment correlation; *r*_E_ = nonshared environment correlation.

Model-fitting analyses were performed that incorporated data at both 9 and 12 years using a bivariate common pathway model (Neale & Maes, 2001). This model decomposed the variance of a latent variable of science performance derived from a phenotypic factor analysis of the different science measures at each age (Figure 1, with confidence intervals included in Table 3). At both 9 and 12 years the individual science measures loaded very highly on the latent variable (average at age 9 = .92; average at age 12 = .96). At age 9, the heritability of the latent variable was high (64%) and shared environmental influences were modest (16%). At age 12, heritability was lower (47%) and shared environmental influences accounted for more of the variance (32%). These results were consistent with the ACE estimates derived from the twin correlations shown in Table 2. Ninety-five percent confidence intervals for these estimates are shown in Table 3; the nonoverlapping confidence intervals indicate that changes from 9 to 12 years were significant for both heritability and shared environment. We also tested this formally by fitting univariate common pathway models at 9 and then at 12 years, and compared the fit of the model when we equated the ACE parameters across these models. There was a significant decrease in fit when we equated the genetic (chi-square difference = 19.816; *p* < .001) and the shared environmental parameters (chi-square difference = 21.698; *p* < .001). We found no significant decrease in fit when we equated the nonshared environmental parameters (chi-square difference = 0.820; *p* = .365).

**Table 3:** Estimates (and 95% Confidence Intervals) From Common Pathway Model for Science at 9 and 12 Years

	Common			
Measures	A	C	E	
9-year Science	.64 (.57–.70)	.16 (.09–.22)	.20 (.19–.22)	
12-year Science	.47 (.42–.51)	.32 (.28–.36)	.21 (.20–.23)	
	*r*_A_	*r*_C_	*r*_E_	*r*_P_
9–12 year Science	.50 (.39–.60)	.78 (.56–.99)	.02 (.00–.11)	.45 (.43–.48)
	A (*a*_*x*_*a*_*y*_*r*_A_/*r*_P_)	C (*c*_*x*_*c*_*y*_*r*_C_/*r*_P_)	E (*e*_*x*_*e*_*y*_*r*_E_/*r*_P_)	
Mediation of *r*_P_	.60 (.47–.73)	.39 (.27–.49)	.01 (.00–.04)	
	Specific			
	A	C	E	Factor loading
9 year				
Scientific enquiry	.02 (.01–.02)	.02 (.02–.03)	.14 (.13–.15)	.88 (.87–.88)
Life processes	.03 (.01–.03)	.04 (.03–.04)	.07 (.06–.07)	.93 (.93–.93)
Physical processes	.04 (.01–.04)	.04 (.03–.04)	.05 (.04–.06)	.96 (.96–.96)
12 year				
Scientific enquiry	.08 (.06–.08)	.03 (.03–.04)	.09 (.08–.09)	.93 (.92–.93)
Life processes	.10 (.07–.11)	.04 (.03–.04)	.05 (.05–.05)	.96 (.96–.96)
Physical processes	.12 (.07–.12)	.04 (.04–.05)	.04 (.04–.05)	.97 (.97–.97)
Materials and properties	.12 (.07–.12)	.05 (.04–.05)	.04 (.04–.04)	.98 (.98–.98)

*Note.* A = additive genetic; C = shared environment; E = nonshared environment; *r*_A_ = genetic correlation; *r*_C_ = shared environmental correlation; *r*_E_ = nonshared environmental correlation; *r*_P_ = phenotypic correlation; A (*a*_*x*_*a*_*y*_*r*_A_/*r*_P_) = proportion of the phenotypic correlation accounted for by genetic influences; C (*c*_*x*_*c*_*y*_*r*_C_/*r*_P_) = proportion of the phenotypic correlation accounted for by shared environmental influences; E (*e*_*x*_*e*_*y*_*r*_E_/*r*_P_) = proportion of the phenotypic correlation accounted for by nonshared environmental influences.

As well as decomposing the variance at each age into genetic and environmental influences, it is possible to decompose the covariance between 9 and 12 years by including the data from both ages in the same model. Here we present the results from this analysis of the covariance between 9 and 12 years as genetic and shared environmental correlations between the latent genetic and environmental factors at 9 and 12 years (Figure 1 and Table 3). The genetic correlation (*r*_A_) between 9 and 12 years was moderate (.50). The shared environmental correlation was high (.78) and the nonshared environmental correlation was insignificant (.02). The phenotypic correlation between the latent variables at 9 and 12 years was .45 (95% confidence intervals for all estimates shown in Table 3). Using this information it is possible to estimate the proportion of the phenotypic correlation between 9 and 12 years that can be accounted for by genetic and environmental factors. That is, to what extent do genetic and environmental factors contribute to the stability in science performance from 9 to 12 years? Genetic influences accounted for 60% of the cross-age continuity in performance, and shared environmental factors accounted for 39% of the continuity.

Finally, the model estimated specific genetic and environmental influences on the remaining variance in the separate science measures. The phenotypic factor analysis indicated that the science measures at each age loaded highly on the latent variable, meaning that there was little residual variance to decompose. We present unstandardized estimates for the specific genetic and environmental influences in Table 3; all of these estimates were small.

## Discussion

In contrast to general cognitive ability and to other academic abilities such as reading and mathematics, the heritability of science performance decreases between middle childhood and early adolescence and the influence of the shared environment increases. Science performance at 9 years was highly heritable (64%) and showed modest influence of the shared environment (16%). At 12 years the heritability of science was significantly lower (47%) and shared environmental influences explained significantly more of the variance (32%). The influence of the nonshared environment was similar at 9 and 12 years (20% and 21%, respectively).

Our findings for science performance at 12 years are compatible with results from the only other study looking at science-related phenotypes. Loehlin and Nichols (1976) studied the genetic and environmental origins of reading comprehension of passages involving social science and natural science in a sample of bright high school students. Heritability was estimated as 40%, and shared environmental influences were of a similar magnitude (30%) to those found for the 12-year olds in this study. The results from the study by Loehlin and Nichols and our study suggest that the shared environment—that is, environmental influences that make members of a twin pair more similar—may be particularly important for science performance in adolescence, as discussed later.

### Developmental Decreases in Heritability

In contrast to the high heritability found at 9 years, the heritability of science performance at 12 years was significantly lower (as indicated by non-overlapping confidence intervals for the two estimates; see Table 3; and significant worsening of fit after equating the estimates at 9 and 12 years). Our longitudinal analysis provided some insight into why heritability might decrease. The genetic correlation of 0.50 between 9 and 12 years suggests that genetic factors that affect science performance at 9 and 12 years are substantially different. (The genetic correlation can be interpreted loosely to mean that genes associated with science performance at 9 years will only have about a 50% chance of being associated with science performance at 12 years.) Thus, the simplest explanation of why heritability decreases is that different genes affect science performance at 9 and 12 years and that the genes that affect science performance at 12 years have less impact.

If the genetic correlation between 9 and 12 years had been very high, the change in heritability could be explained by genotype–environment correlation. Increases in heritability during development for general cognitive ability (McGue et al., 1993) are typically attributed to genotype–environment correlation (Bergen, Gardner, & Kendler, 2007; Plomin, DeFries, & Loehlin, 1977; Scarr & McCartney, 1983); that is, individuals seek out environments that are correlated genetically with the phenotype. Speculation as to why genotype–environment correlation might operate in the opposite direction for science in adolescence includes the possibility that young people are not being motivated to engage in science and therefore are not seeking out scientifically enriching environments correlated with their genetic predilections. In other words, experience in relevant environments, an essential condition for improved performance, may be driven less by genetics and more by environmental influences for science than other domains. There is some evidence for dwindling enthusiasm for school science in early adolescence (Jenkins & Nelson, 2005; Osborne, Simon, & Collins, 2003), but an investigation as to why this happens is needed; for example, puberty is a possible mechanism for these decreases in engagement with education. (The timing of puberty itself is of course influenced by both genetic and environmental factors.) Decreases in the *relative* contribution of genetics to performance might also be due to increases in environmental variance.

### Increasing Importance of Shared Environment

Shared environmental influences account for significantly more variance at 12 years than at 9 years. What are the possible mechanisms for this change? Again, our longitudinal analyses help to frame the question. The correlation between shared environmental influences at 9 and 12 years is 0.78, substantially greater than the genetic correlation of 0.50. This high shared environmental correlation suggests that the same shared environmental factors affect science performance at 9 and 12 years. Thus, the increasing shared environmental influence from 9 to 12 years suggests that although the same shared environmental factors might affect science performance at 9 and 12 years they have more of an effect at 12 years.

The obvious candidate for shared environment is the educational experience that which is shared because the two twins attend the same schools. Between 9 and 12 years, the twins transfer from Key Stage 2 to Key Stage 3 of the NC; in addition, many of the twins transfer from primary to secondary school. Both of these factors could produce differences in the etiology of science performance, in particular, by increasing variance in environmental experiences.

The present results clearly demonstrate an unexpected pattern: Unlike other academic abilities, shared environmental factors are increasingly important for science performance. Following these twins into late adolescence and early adulthood will help to clarify these processes, as will the collection of information regarding the science classroom environment. It will be important to identify these environmental influences as they may lead to interventions that encourage more individuals to engage with science.

### Nonshared Environmental Influences on Science Performance

Nonshared environmental influences are environmental influences that do not contribute to within-pair similarity for a trait. These could be objectively “nonshared” experiences such as having different friends or experiencing an accident. However, they may also include an individual’s perception of the environment. Even MZ twins who are genetic clones of one another experience the same classroom environment differently (Asbury, Almeida, Hibel, Harlaar, & Plomin, 2008). For science performance at both 9 and 12 years, nonshared environmental influences explain a significant proportion of the variance (20% and 21%). Although nonshared environment also includes measurement error, using the latent variable approach reduces the error variance included in our latent science variable. Because nonshared environmental influences are the only source of variance that make MZ twins different from one another, a powerful design for assessing nonshared experiences is the MZ differences design. In a subsample of TEDS we have recently shown that MZ differences in school positivity and flow in the science classroom can explain some of the differences in science performance (Asbury et al., 2008).

As well as being unique to each member of a twin pair, nonshared environmental influences are specific to science performance at each age, with no significant overlap in nonshared environmental influences from 9 to 12 years. Similar results are found for reading and mathematics (Kovas, Petrill, et al., 2007). Nonshared environmental influences may therefore incorporate many types of age-specific experiences such as the influence of teacher and peer relationships. Whatever these influences are they do not contribute to developmental stability in performance.

In early adolescence as well as moving to secondary schools, students start making decisions about their own education and their future, searching for more autonomy in their learning, as well as for positive and supportive relationships with peers and nonparental adults (Ryan & Patrick, 2001). Environments that are sensitive to the changing role of the student in the classroom during adolescence are associated with more positive student outcomes, and those classrooms that are not sensitive to these needs have produced more negative results (Midgley & Feldlaufer, 1987; Midgley, Feldlaufer, & Eccles, 1989). Peer relationships are also important in the classroom and can promote engagement with academic achievement (Berndt & Keefe, 1995; Brown, 1990; Parker & Asher, 1987). These are examples of possible targets for nonshared environmental influence.

### Limitations

An apparent limitation of this study is the use of teacher-report data rather than objectively measured science performance. However, such teacher reports are an important part of the U.K. NC and may actually represent a more comprehensive and valid assessment of the child’s ability in science over the course of a year than a single test. A major emphasis in school science is to foster scientific enquiry and reasoning; such skills may be difficult to assess in formal testing. Moreover, much of the previous work on academic abilities has focused on teacher-report data, an approach that is justified by the generally high correlations between objective test data and teacher reports that have been found (see, e.g., Harlaar, Dale, & Plomin, 2005b). Nonetheless, we intend to complement our teacher reports of science performance with an objective test of performance when the twins are 14 years old.

Although the twin method in general has its limitations, research into the assumptions of the twin method have consistently found that they are reasonably well met; for this reason the twin method has been used throughout the medical and behavioral sciences as a rough estimate of the influence of nature and nurture (e.g., Martin, Boomsma, & Machin, 1997). It might also be considered a limitation that we are using the twin method as a rough guide to the relative influence of nature and nurture rather than conducting molecular genetic research to identify specific differences in DNA sequence that are responsible for heritable differences between individuals. However, it is expensive and difficult to identify even some of the many DNA differences likely to be responsible for any common disorders or complex traits (Plomin, Kennedy, & Craig, 2006). Therefore it is a more productive strategy to investigate etiology within a quantitative genetic design, and then to use this information to inform the design of molecular genetic studies. For instance, given that the different components of science performance are so highly correlated, it would be appropriate to investigate a general science factor in molecular genetic research. Another example is the genetic correlation of 0.50 between science performance at 9 and 12 years that suggests that different genes would be found at the two ages. Moreover, unlike molecular genetic research, which is limited to addressing genetics, the twin method provides as much information about the environment as it does about genetic influences. Future work should also include specific measures of environments that may be important for science performance, such as school and family characteristics and science facilities and opportunities.

The phenotypic correlation between science at 9 and 12 years is modest (*r* = .45), even though the content of the science measure is similar at both assessments, and based on the criteria of the U.K. NC. We cannot rule out the possibility that the modest phenotypic correlation may reflect changes in what has been measured; neither can we say whether this change reflects genuine developmental changes in understanding of science and the cognitive processes needed for science. As noted below, both measurements showed good reliability, and therefore the change across time could reflect genuine developmental changes.

Finally, one potential limitation is that at 12 years most of the twins are in a new school, where teachers may not know them as well and subsequently the measure may be less reliable. The MZ twin correlation is a good index of reliability, as the only influence that makes MZ twins less similar is the nonshared environment that also includes measurement error. A decrease in the MZ correlation and therefore an increase in the nonshared environment could be a sign that the measure is less reliable. In this study the MZ correlations were almost identical at the two ages (averages = .74 and .73), suggesting that the measure at 12 years is just as reliable as the measure at 9 years. As the twins move from primary to secondary school there may be a change in the degree to that they share their classroom experience. At 9 years 63% of the twins were in the same classroom, and we have shown that the effect of same teacher versus different teachers has little effect on our ACE estimates (Kovas, Petrill, et al., 2007). However, at 12 years we do not have information on whether the twins share a science teacher. We therefore cannot rule out the possibility that some of the twins will have moved from having a different teacher to sharing the same teacher, but also some twins will go from having the same teacher to having a different teacher. We can also not rule out the possibility that these results are twin specific—in that twins may work together more as the curriculum gets more challenging—but we are unaware of any empirical evidence of this. In summary, we cannot specify the mechanisms for our findings of decreased heritability and increased shared environment but will be able to test this more formally when specific genes and environments for science performance are identified.

### Conclusions

For school science performance, shared environmental influences become significantly more important as children progress through school, and genetic influences become less important. Future work should attempt to identify the specific genes and specific environmental factors that influence science performance. Environmental influences for science become more important during adolescence; an understanding of what these environmental influences are could provide insights into educational interventions that promote interest and achievement in science throughout the lifespan.
